# Circulating miRNA-195-5p and -451a in Patients with Acute Hemorrhagic Stroke in Emergency Department

**DOI:** 10.3390/life12050763

**Published:** 2022-05-21

**Authors:** Mauro Giordano, Maria Consiglia Trotta, Tiziana Ciarambino, Michele D’Amico, Federico Schettini, Angela Di Sisto, Valentina D’Auria, Antonio Voza, Lorenzo Salvatore Malatino, Gianni Biolo, Filippo Mearelli, Francesco Franceschi, Giuseppe Paolisso, Luigi Elio Adinolfi

**Affiliations:** 1 Department of Advanced Medical and Surgical Sciences, University of Campania “Luigi Vanvitelli”, 80138 Naples, Italy; fede.skett@gmail.com (F.S.); angeladis90@gmail.com (A.D.S.); valentinadauria84@gmail.com (V.D.); giuseppe.paolisso@unicampania.it (G.P.); luigielio.adinolfi@unicampania.it (L.E.A.); 2 Study and Research Center of the Italian Society of Emergency Medicine (SIMEU), 10155 Turin, Italy; antonio.voza@humanitas.it (A.V.); malatino@unict.it (L.S.M.); francesco.franceschi@unicatt.it (F.F.); 3 Department of Experimental Medicine, Division of Pharmacology, University of Campania “Luigi Vanvitelli”, 80138 Naples, Italy; mariaconsiglia.trotta2@unicampania.it (M.C.T.); michele.damico@unicampania.it (M.D.); 4 Department of Internal Medicine, Hospital of Marcianise, ASL Caserta, 81025 Caserta, Italy; tiziana.ciarambino@gmail.com; 5 Emergency Department, Humanitas Research Hospital, 20089 Milan, Italy; 6 Department of Clinical and Experimental Medicine, University of Catania, 95126 Catania, Italy; 7 Department of Medical and Surgical Sciences, University of Trieste, 34149 Trieste, Italy; biolo@units.it (G.B.); filippome@libero.it (F.M.); 8 Department of Emergency Medicine, Fondazione Policlinico Universitario A. Gemelli IRCCS, Università Cattolica del Sacro Cuore, 00168 Roma, Italy

**Keywords:** microRNA, intracerebral hemorrhagic stroke, acute ischemic stroke, emergency

## Abstract

(1) Background: In our previous study, acute ischemic stroke (AIS) patients showed increased levels of circulating miRNAs (-195-5p and -451a) involved in vascular endothelial growth factor A (VEGF-A) regulation. Here, we evaluated, for the first time, both circulating miRNAs in acute intracerebral hemorrhagic (ICH) patients. (2) Methods: Circulating miRNAs and serum VEGF-A were assessed by real-time PCR and ELISA in 20 acute ICH, 21 AIS patients, and 21 controls. These were evaluated at hospital admission (T0) and after 96 h (T96) from admission. (3) Results: At T0, circulating miRNAs were five-times up-regulated in AIS patients, tending to decrease at T96. By contrast, in the acute ICH group, circulating miRNAs were significantly increased at both T0 and T96. Moreover, a significant decrease was observed in serum VEGF-A levels at T0 in AIS patients, tending to increase at T96. Conversely, in acute ICH patients, the levels of VEGF-A were significantly decreased at both T0 and T96. (4) Conclusions: The absence of a reduction in circulating miRNAs (195-5p and -451a), reported in acute ICH subjects after 96 h from hospital admission, together with the absence of increment of serum VEGF-A, may represent useful biomarkers indicating the severe brain damage status that characterizes acute ICH patients.

## 1. Introduction

Acute intracerebral hemorrhagic stroke (ICH) can be considered one of the major neurological disorders contributing to global risk of morbidity and mortality [1,2]. Indeed, even if it accounts for the 10–15% of total stroke events, its incidence progressively increases with age [3,4,5,6].

Although the recent research progress in acute ICH physiopathology identified microvascular disorders (such as cerebral arteriopathy or amyloid angiopathy) as the predominant factors leading to non-traumatic bleeding within the brain parenchyma [1], acute ICH management is still challenging. The current medical drugs considered to be the of standard care in acute ICH treatment aim to obtain the reversal of coagulopathy, as well as the control of blood and intracranial pressure [7,8]. However, these are not associated with significant clinical and functional improvements [2]. Therefore, the development and validation of different ICH prognostic models, along with the research of innovative therapeutic strategies for preventing hematoma expansion or favoring hematoma evacuation with low invasive methods, is considered to be of great interest for benefitting ICH patients [2,7]. 

In this context, the identification of serum biomarkers profiles, following a stroke event, could substantially contribute to identify the severity of brain damage and its evolution. In this regard, previous studies have proposed different serum mediators as possible clinical biomarkers of acute stroke, such as circulating erythropoietin [9], inflammatory markers (C-reactive protein, interleukin 6, and fibrinogen) [10,11], N-terminal–pro-B-type natriuretic peptide and endostatin [11,12], BDNF [13], and VEGF [14]. However, none of these circulating mediators were able to provide an accurate diagnosis differentiating AIS from an acute ICH stroke event. This is a critical issue for the immediate and effective management of an acute cerebrovascular accident in the early phases, in order to differentiate between the therapeutic strategies [8]. Indeed, it has to be considered that there are substantial differences between AIS and ICH cohorts, with regard to neurological severity and stroke recovery. In fact, was reported in a recent analysis on more than 180,000 acute stroke patients that the short-term, functional outcomes of hospital admission were improved mainly in AIS patients, presumably due to the efficacy of reperfusion therapy, while they were almost absent in ICH group [15]. 

Therefore, a great effort has been made to identify the promising candidates that are differentially expressed by AIS and acute ICH patients, in order to have an early and non-invasive diagnosis of acute stroke subtypes. Among them, the most suitable serum or plasmatic predicted biomarkers are S100β, ubiquitin carboxyterminal hydrolase-L1, glial fibrillary acidic protein, retinol-binding protein 4, and a soluble receptor for an advanced glycation end product [11,16,17,18,19,20,21]. 

In this regard, circulating miRNAs have been analyzed as potential sensitive biomarkers of a specific acute stroke subtype. Particularly, several clinical studies reported different serum miRNAs that are specifically associated with AIS [22,23,24,25,26,27,28,29,30,31,32,33], as well as different circulating miRNA levels that correlate with ICH [34,35,36,37,38,39]. The latter is mainly involved in the regulation of hematoma and perihematomal edema, as well as endothelial dysfunction [34]. However, to our knowledge, no studies reported the same circulating miRNA as differentially expressed between AIS or ICH patients. In particular, previous studies have suggested the key roles of several microRNAs (miRNAs) in post-ischemic angiogenesis, achieved by acting on specific targets aimed at restoring blood supply after an ischemic stroke [40,41,42,43,44,45]. Indeed, as small non-coding RNAs are able to silence gene expression, miRNAs may regulate the levels of different mediators acting in post-stroke angiogenesis and vascular angiogenic remodeling [46,47]. Among the miRNAs pattern, several of them have been reported to be dysregulated in the serums of patients with acute stroke [22,48,49]. Thanks to their high stability and easy detection, circulating miRNAs may reflect the underlying stroke pathophysiological mechanisms and post-stroke clinical consequences [22].

We previously reported on the high circulating miRNA-195-5p and miRNA-451a levels after ischemic stroke in both diabetic and non-diabetic patients in emergency departments. Data support the possible role of hypoxia in regulating both miRNA expressions, which were two-fold up-regulated in diabetic acute ischemic stroke (AIS) and transient ischemic attack patients, compared to non-diabetics, and inversely correlated with both brain-derived neurotrophic factor (BDNF) and vascular endothelial growth factor A (VEGF-A) serum levels [23,24]. Particularly, serum VEGF-A was significantly reduced during the early phase in AIS patients, and its levels tend to increase over periods ranging from hours to days post-stroke [23], inversely paralleling the decrease of miRNA-195-5p and miRNA-451a levels. Furthermore, the importance of post-stroke angiogenesis and VEGF-A has been reported in both animals and human studies with stroke [50,51,52,53,54,55].

Indeed, vascular remodeling mediators, such as VEGF-A, are strictly involved in brain recovery and circulation after stroke [56,57,58,59,60]. They seem to be involved in increasing the oxygen supply to the boundary zone by avoiding brain tissue necrosis; then, they furnish nutrients by promoting the generation of new neurons and synapses [61]. These pathophysiological mechanisms in post-stroke angiogenesis are particularly crucial in the recovery from acute intracerebral hemorrhage stroke (ICH) [1], characterized by non-traumatic bleeding and formation of a hematoma in the brain parenchyma [3,62]. 

In this scenario, no evidence has been reported on the possible changes in serum VEGF-A levels in IHC patients; additionally, no data have been reported regarding the levels of circulating miRNAs (-195-5p and -451a) in ICH patients.

Thus, we evaluated, for the first time, both circulating miRNAs expression and serum VEGF-A levels in ICH patients, in comparison with AIS patients, at two different time points (at hospital admission and after 96 h from admission). We have also evaluated, in both acute ICH and AIS patients, whether miRNA-195-5p and miRNA-451 expression could correlate with VEGF-A levels. 

## 2. Materials and Methods

### 2.1. Selection of Participants

The present study was performed at the Hospital of Marcianise, University of Campania, “Luigi Vanvitelli”, Italy. A total of 41 stroke patients (21 with ICH and 20 with AIS) were included in the study, together with 21 patients with no history of cerebrovascular diseases or previous ischemic stroke (control group, C). All study groups were matched for age and sex, including the controls. All patients had moderate to severe strokes, based on a National Institutes of Health Stroke Scale (NIHSS) score between 5–20. ICH was defined as an episode of primary, spontaneous, non-traumatic bleeding occurring in the brain parenchyma [7], based on noncontrast computerized tomography (NCCT), the gold standard technique used for a fast and sensitive diagnosis of ICH [2,63]. AIS was defined as an episode of acute neurological dysfunction caused by focal cerebral ischemia, based on objective imaging techniques, such as CT and clinical evidence of cerebral focal ischemic injury, based on symptoms of any duration, as described in a previous study [24].

The inclusion criterion for this study were the following: presentation in our emergency department after 4.5 h of symptom onset or recognition (ineligible for IV thrombolysis) [64]; NIHSS score between 5–20; modified Rankin scale between 3–4 [65]; age older than 60 years; APACHE II score evaluated lower than 22; and Cincinnati score positive for presence of neurological symptoms at hospital admission, confirmed by neuroimaging evaluations [66]. The following exclusion criteria were considered: body temperature higher than 37.5 °C; history of cancer; history of surgery within 6 months; severe anemia (Hgb < 7.5 g/dL); acute arrhythmias; acute coronary disease; or participation in other clinical studies. All patients signed an informed consent. The study was approved by Ethical Review Board of North Campania, Italy (CECN/802, 7 February 2018).

### 2.2. Interventions

Serum samples were obtained from the patients’ blood at admission (T0) and after 96 h (T96) from hospital admission, in order to evaluate circulating miRNAs (195-5p and -451a), along with VEGF-A serum levels, in acute ICH and AIS patients. 

### 2.3. miRNA Isolation and Real-Time Reverse Transcription (qRT-PCR)

miRNA extraction, quantization, and reverse-transcription to cDNA were performed as previously described [23,24]. Particularly, starting from a serum volume of 200 µL, miRNA isolation was performed by using the miRNeasy Serum/Plasma kit (Qiagen, Hilden, Germany), according to the manufacturer’s protocol for miRNA purification from human serum. Specifically, sample lysis was obtained by homogenization in a specific phenol/guanidine thiocyanate monophasic solution provided by the kit (QIAzol Lysis Reagent, Hilden, Germany). Before the addition of chloroform, in order to obtain the separation between organic phases (containing DNA and proteins) and aqueous phases (containing RNA) following centrifugation, Syn-cel-miR-39 miScripit miRNA Mimic 5 nM (Qiagen) was spiked in each sample and used as external control for both extraction and data quantization. After centrifugation, a proper volume of ethanol was added to the upper aqueous phase containing the RNA. The samples were then applied on RNeasy Mini spin columns (Qiagen), in order to let the total RNA (containing miRNAs) bind to the column membrane and wash away all the contaminants before RNA elution in RNase-free water. miRNAs were then converted to cDNA using the miScript II RT kit (Qiagen), according to the manufacturer’s protocol for specific and sensitive reverse transcription of mature miRNAs. Hsa-miRNA-195-5p, hsa-miRNA-451a, and Syn-cel-miR-39 expression levels were detected by real-time PCR analysis using the miScript SYBR Green PCR kit (Qiagen), in combination with specific miScript primer assays (Qiagen). Triplicate determinations were carried out on the CFX96 Real-Time System C1000 Touch Thermal Cycler (BioRad Laboratories, Inc., Hercules, CA, USA). Analyses of the Ct values were performed with the CFX Manager^TM^ Software (BioRad Laboratories, Inc.), while the relative quantification of the miRNA levels was carried out by using the 2^−^^ΔΔCt^ method. 

### 2.4. Serum VEGF-A ELISA Assay

VEGF-A levels were measured using the VEGF-A Human ELISA kit (BMS277-2), Thermo Fisher Scientific, Waltham, WA, USA), following the manufacturer’s instructions.

### 2.5. Outcomes

Serum levels were observed for miRNA-195-5p, miRNA-451a and VEGF-A in patients with IHC or AIS upon admission (T0) and after 96 h (T96). 

### 2.6. Statistical Analysis

Data are reported as mean ± standard error of the mean (S.E.M.) and analyzed by using repeated measures of two-way analyses of variance (ANOVA), followed by Tukey’s multiple comparison test. For both the qRT-PCR and ELISA evaluations, three independent experiments were performed. Pearson correlation analysis was used for the determination of the associations between the VEGF-A and miRNA levels. A probability of *p* < 0.05 was considered sufficient to reject the null hypothesis for all the results. 

## 3. Results

### 3.1. Characteristics of Study Subjects

The clinical characteristics in the control, ICH, and AIS are reported in Table 1. No difference was reported between age and sex in the study groups. 

### 3.2. Circulating miRNA-195-5p in ICH and AIS Patients

**Figure 1:**
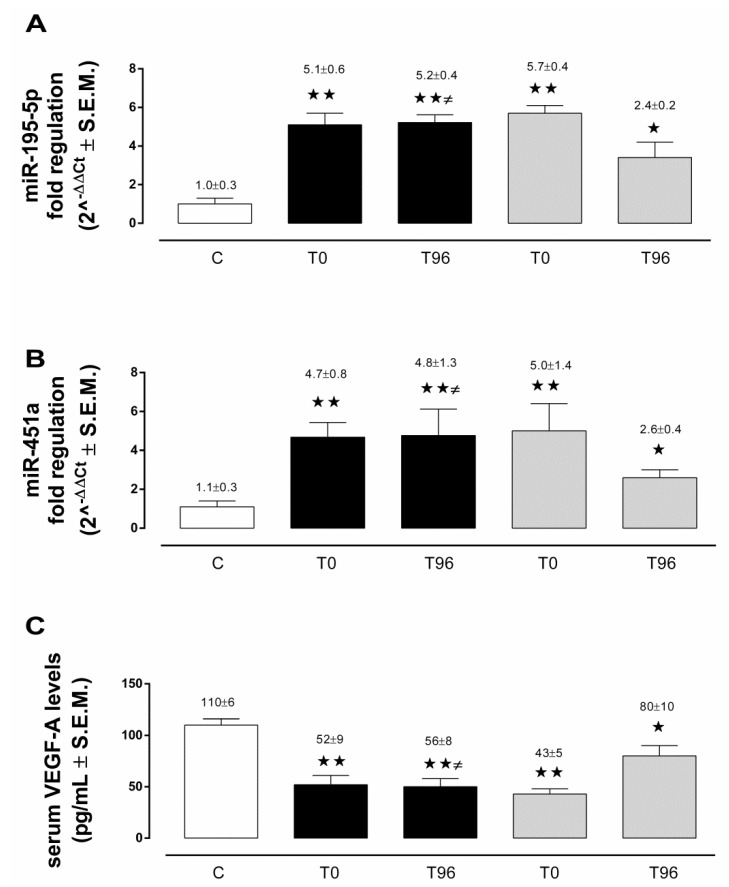
Serum levels of miR-195-5p (**A**), miR-451a (**B**), and vascular endothelial growth factor A (VEGF-A) (**C**) in the control subjects (C) (white column), acute intracerebral hemorrhage stroke patients (ICH) (black column), and acute ischemic stroke patients (AIS) (grey column) at admission (T0) and after 96 h (T96) from admission. miRNA levels are reported as the mean of 2^−ΔΔCt^ ± S.E.M.; VEGF-A levels are reported as pg/mL ± S.E.M. ★ *p* < 0.05 vs. C; ★★ *p* < 0.01 vs. C; ≠ *p* < 0.05 vs. AIS at T96.

At admission (T0), circulating miRNA-195-5p levels were significantly up-regulated in both ICH (2^−ΔΔCt^ = 5.1 ± 0.6; *p* < 0.01 vs. C) and AIS (2^−ΔΔCt^ = 5.7 ± 0.4; *p* < 0.01 vs. C) patients, compared to the control subjects (2^−ΔΔCt^ = 1.0 ± 0.3). After 96 h from admission (T96), while circulating miRNA-195-5p levels were markedly decreased in AIS patients (2^−ΔΔCt^ = 2.4 ± 0.2; *p* < 0.05 vs. C), these were still significantly up-regulated in ICH patients (2^−ΔΔCt^ = 5.2 ± 0.4; *p* < 0.01 vs. C; and *p* < 0.05 vs. AIS), in comparison to both the C and AIS groups (Figure 1A).

### 3.3. Circulating miRNA-451a in ICH and AIS Patients

Similarly, circulating miRNA-451a levels were significantly up-regulated at T0 in both ICH (2^−ΔΔCt^ = 4.7 ± 0.8; *p* < 0.01 vs. C) and AIS (2^−ΔΔCt^ = 5.0 ± 1.4; *p* < 0.01 vs. C) patients, in comparison to C (2^−ΔΔCt^ = 1.1 ± 0.3). At T96, AIS patients showed a significant reduction of circulating miRNA-451a levels (2^−ΔΔCt^ = 2.6 ± 0.4; *p* < 0.05 vs. C), which was absent in ICH patients (2^−ΔΔCt^ = 4.8 ± 1.3; *p* < 0.01 vs. C; and *p* < 0.05 vs. AIS) (Figure 1B).

### 3.4. Serum VEGF-A in ICH and AIS Patients

At T0, serum VEGF-A levels were significantly lower in ICH (52.0 ± 9.0 pg/mL; *p* < 0.01 vs. C) and AIS (43.0 ± 5.0 pg/mL; *p* < 0.01 vs. C) patients, compared to C (110.0 ± 6.0 pg/mL). While serum VEGF-A levels tended to increase in AIS patients at T96 (80.0 ± 10.0 pg/mL; *p* < 0.05 vs. C), these were significantly lower in ICH patients (56.0 ± 8 pg/mL; *p* < 0.01 vs. C; and *p* < 0.05 vs. AIS) (Figure 1C).

### 3.5. Correlation between Circulating miRNAs and VEGF-A Serum Levels

**Figure 2:**
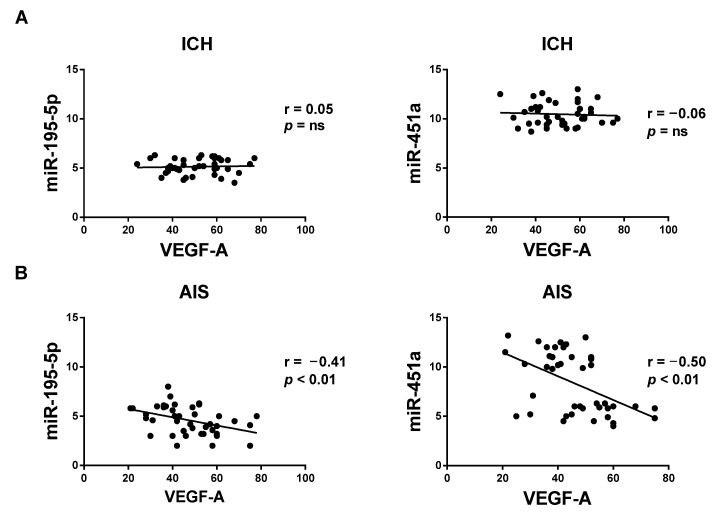
Correlation between serum levels of miR-195-5p and -451a (2^−ΔΔCt^) and vascular endothelial growth factor A (VEGF-A; pg/mL) in (**A**) acute intracerebral hemorrhage stroke patients (ICH; both ns) and (**B**) acute ischemic stroke patients (AIS) (both *p* < 0.01). ns = not significant.

In the ICH population, circulating miRNA-195-5p and -451a levels (2^−ΔΔCt^), up to 96 h from admission, were not significantly associated with serum VEGF-A levels (pg/mL) (Figure 2A).

On the contrary, a significant negative correlation was observed between both the circulating miRNA-195-5p and -451a expression and VEGF-A serum levels (miR-195-5p: r = −0.41, *p* < 0.01; miR-451a: r = −0.50, *p* < 0.01) (Figure 2B). 

## 4. Discussion

In the present study, we report a significant increase in circulating miRNA-195-5p and -451a expression in acute ICH patients. Several studies have shown an involvement of these two miRNAs during a hemorrhage in both animal and human studies [67,68]. In particular, a role of miRNA-195-5p and -451a has been reported, and a modulation of their expression has been hypothesized to be a useful tool for neoangiogenesis and neurogenesis [67,68] for cerebral tissue recovery after hemorrhagic stroke. These two miRNAs seem to be related to changes in the expression levels of the pro-angiogenic factor VEGF-A [23,24,69,70]. In addition, it seems that the high expression of these miRNAs relates to low VEGF-A and, thus, putative angiogenesis [23,24,69,70]. In line with this concept, the present study reports a significant increase in circulating miRNA-195-5p and -451a expression in acute ICH.

Of interest, the study reports, for the first time, that the levels of circulating miRNAs-195-5p and -451a in ICH patients did not change after 96 h, underlying a persistent impaired angiogenesis in these patients. In this regard, we have previously reported that AIS patients had a higher expression of circulating miRNA-195-5p and miRNA-451a [23,24], both of which were associated with VEGF-A regulation [69,70]. We previously reported a significant decline (within 72 h) of these circulating miRNAs in AIS patients, which correlated to the increment of VEGF-A levels, suggesting an attempt to recover from brain vascular damage [23,24]. In line with this trend, in the present study, we also observed a significant circulating miRNA reduction in AIS patients after 96 h. In addition, we also reported, for the first time, that circulating miRNA (-195-5p and -451a) levels in ICH patients did not change after 96 h. To date, we do not know the exact reason that such a difference was observed between ICH and AIS strokes. However, it cannot be excluded that the absence of a reduction of miRNA in ICH patients after 96 h may represent the result of the hemorrhage-induced brain damage, not followed by a gradual functional recovery, as reported in AIS patients. In fact, we did not observe an incremental change in serum VEGF-A levels after 96 h in ICH patients, as compared to what was observed in AIS patients. These findings could be explained as a possible expression of the vascular damage induced by the hemorrhage. Unfortunately, no study has previously evaluated such a condition over time in ICH patients. 

In particular, we observed a significant negative linear correlation (r = −0.41, *p* < 0.01 for miRNA-195-5p and r = −0.50, *p* < 0.01 for miRNA-451a) between the serum miRNA-195-5p and miRNA-451a expression vs. serum VEGF-A levels in AIS patients. However, no correlation was found between the circulating miRNA expression and serum VEGF-A levels in ICH patients (*p* = ns). These data may suggest a different response over time (96 h) between ICH and AIS patients on the recovery from vascular brain damage. In this regard, future studies may highlight the utility of evaluating these serum mediators (miRNAs ad VEGF-A) as possible biomarkers for better understanding the evolution of vascular and brain damage severity. 

## 5. Conclusions

These data shows, for the first time, that circulating miRNAs (-195-5p and -451a) levels in acute ICH patients did not change after 96 h from emergency hospital admission, as compared to what was observed in AIS patients. Our results may also indicate the important role of these miRNAs in differentiating ICH patients from AIS patients. Furthermore, the incremental change of VEGF-A in AIS patients observed after 96 h was not present in the ICH patients. The absence of the reduction in circulating miRNAs (195-5p and -451a) was reported in ICH patients, together with the absence of incremental change of VEGF-A, may represent useful biomarkers, indicating the severity of the vascular brain damage that characterizes ICH patients. Although miRNA 195-5p and 451 have been reported recently as potentially helpful in the evaluation of vascular brain damage, especially between ICH and AIS patients, as reported in the present study, we are not aware of a validation study on these genes; future studies are needed, in order to validate such miRNAs as potential clinical stroke biomarkers. The main limitations of the study are represented by the small groups of patients in a single center. However, this is a “proof of concept” study, opening the door to further, larger studies to confirm our results. 

## Figures and Tables

**Table 1:** Clinical characteristics in control (C), acute intracerebral hemorrhage stroke (ICH), and acute ischemic stroke (AIS) groups. N (total number), M (number of males), BMI (body mass index), systolic blood pressure (SBP), and diastolic blood pressure (DBP). The values are indicated by percentage and mean ± S.E.M.; ns (not significant vs. C); ns * (not significant vs. AIS).

	C	ICH	AIS	*p* Value
N (M)	21 (10)	21 (11)	20 (9)	ns
Age (years)	69 ± 2	68 ± 3	73 ± 5	ns
BMI (kg/m^2^)	27 ± 2	25 ± 5	26 ± 4	ns
SBP (mmHg)	142 ± 6	141 ± 7	139 ± 5	ns
DBP (mmHg)	81 ± 2	85 ± 5	82 ± 3	ns
Hypertension (%)	9 (43)	11 (52)	9 (45)	ns
Smoking (%)	(25)	(35)	(35)	ns
Hyperlipidemia (%)	(40)	(45)	(50)	ns
NIHSS score	-	15 ± 1.4	16 ± 1.6	ns *
Modified Rankin scale	-	3.7 ± 0.4	3.5 ± 0.3	ns *

## Data Availability

The data presented in this study are contained within the article.
